# Supplementary material for: Apple Blossom Agricultural Residues as a Sustainable Source of Bioactive Peptides through Microbial Fermentation Bioprocessing
Source: Antioxidants (Basel). 2024 Jul 13;13(7):837. doi: 10.3390/antiox13070837 (PMC11273824; doi:10.3390/antiox13070837)

Figure S1. Separation by HPLC (RI) of carbohydrates, organic acids, and ethanol in raw apple flowers (Raw-AF), AF without microbial inoculum (Unstarted-AF), and Fermented-AF, which were incubated for 24 h at 30 °C. Fermentations (Fermented-AF) were carried out using *Fructobacillus fructosus* PL22 (PL22-AF) and *Wickerhamomyces anomalus* GY1 (GY1-AF).

Peak assignments: 1, Glucose; 2, Fructose; 3, Mannitol; 4, unknown; 5, Lactic acid; 6, Acetic acid; 7, unknown; 8, Ethanol.

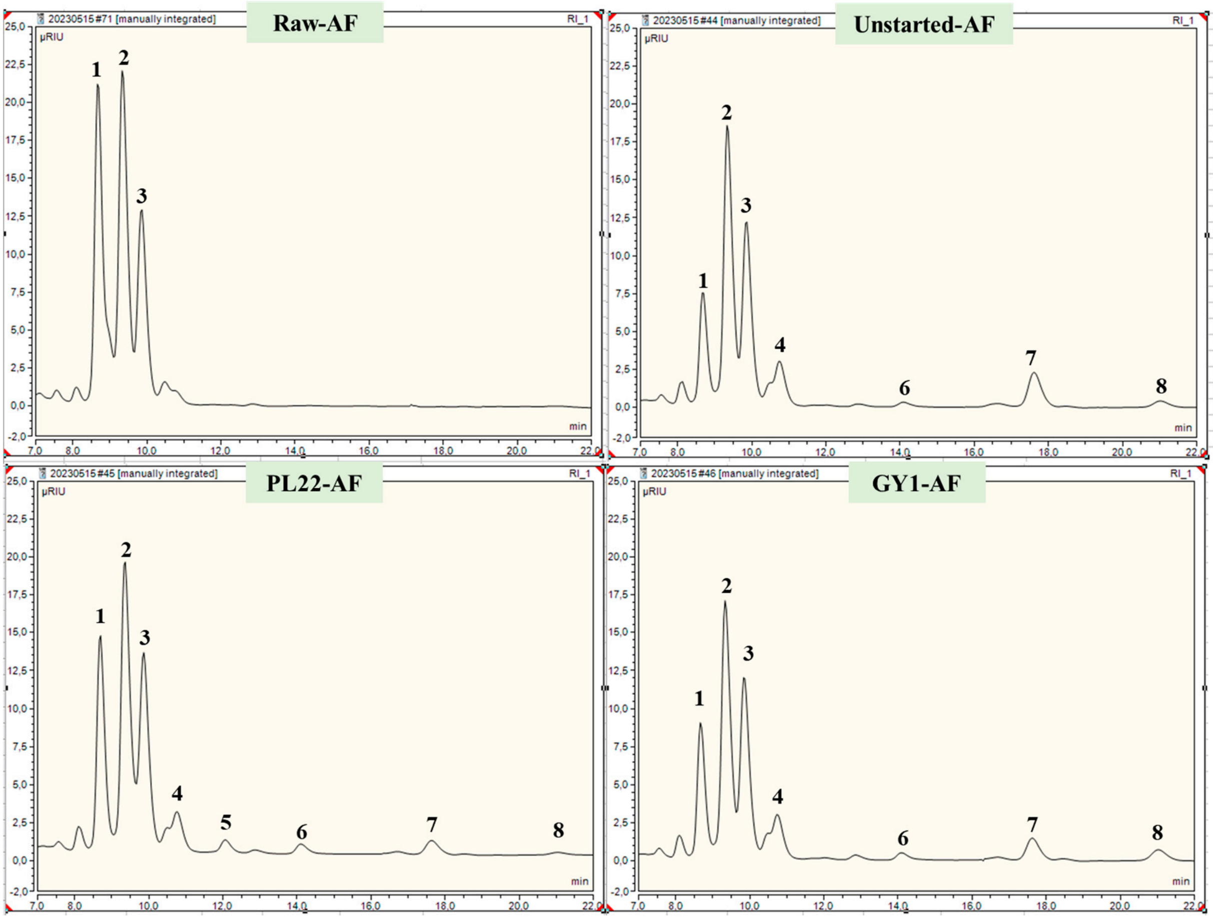

Supplement: Supplementary file 1 [file antioxidants-13-00837-s001.zip › Figure S1.pdf]
